# Abnormal retinal thickening is a common feature among patients with ARSACS-related phenotypes

**DOI:** 10.1136/bjophthalmol-2013-304534

**Published:** 2014-01-23

**Authors:** Patrick Yu-Wai-Man, Angela Pyle, Helen Griffin, Mauro Santibanez-Korev, Rita Horvath, Patrick F Chinnery

**Affiliations:** 1 Wellcome Trust Centre for Mitochondrial Research, Institute of Genetic Medicine, Newcastle University, Newcastle upon Tyne, Tyne and Wear, UK; 2 Department of Ophthalmology, Royal Victoria Infirmary, Newcastle upon Tyne, Tyne and Wear, UK; 3 Department of Neurology, Royal Victoria Infirmary, Newcastle upon Tyne, Tyne and Wear, UK

**Keywords:** Optic Nerve, Genetics

Autosomal-recessive spastic ataxia of Charlevoix-Saguenay (ARSACS) was first described among French Canadian patients from Québec presenting with a stereotypical triad of early-onset cerebellar ataxia, spastic paraplegia and peripheral neuropathy. Two recurring pathogenic mutations in the *SACS* gene were subsequently identified in these families in keeping with a mutational founder event in a geographically isolated population.^1^ However, ARSACS is being increasingly recognised worldwide as an important cause of inherited ataxia.^2^ Interestingly, non-Québec patients can show strikingly variable features marked by a lack of spasticity, cognitive impairment and a delayed age of onset.^2^ Given the heterogeneous clinical picture that can be associated with *SACS* mutations, the identification of ancillary features linked with these genetic defects could prove particularly useful in prioritising the most appropriate lines of investigations when confronted with a suspected case of ARSACS. Although prominent retinal hypermyelination is thought to be a characteristic manifestation of classical ARSACS among Québec patients, this ophthalmological finding has only been described infrequently in patients from Europe, Asia and the Middle East.^2^ A recent case report has even further argued that retinal ‘hypermyelination’ in ARSACS is a pathologically misleading term that should be abandoned in favour of retinal nerve fibre layer (RNFL) hypertrophy.^3^

To more accurately define the nature of the retinal findings in ARSACS and its possible practical relevance as a screening tool in routine clinical practice, we carried out a comprehensive neuro-ophthalmological examination of five patients from the North of England with molecularly confirmed ARSACS (table 1).^4^ ^5^ Topographic analysis of the optic disc was performed with the Spectralis optical coherence tomography (OCT) platform (Heidelberg Engineering, Heidelberg, Germany).

**Table 1:** Clinical features and retinal nerve fibre layer measurements in patients with pathogenic *SACS* mutations

Patient	Age (years)	Sex	Onset (years)	Clinical features	*SACS* mutations	Average RNFL Thickness	
						OD (μm)	OS (μm)
A	48	F	26	Gait ataxia, dysarthria, spastic paraplegia and peripheral neuropathy	c.2076delG (p.Thr692ThrfsX713); c.3965_3966delAC (p.Gly1322ValfsX1343)	174	175
B	45	M	19	Gait ataxia, dysarthria, spastic paraplegia and peripheral neuropathy		162	140
C	43	M	Late-teens	Gait ataxia, dysarthria, proximal myopathy and peripheral neuropathy	c.13048G>T (p.Glu4350X); 0.7Mb deletion (13q12.12)	138	122
D	46	M	Mid-teens	Gait ataxia, dysarthria, proximal myopathy and peripheral neuropathy		152	169
E	69	M	Late-teens	Gait ataxia, spastic paraplegia and peripheral neuropathy	c.1580C>G (p.Ser527X); c.6781C>A (p.Leu2261Ile)	111	86

Patients A and B, and patients C and D, are two pairs of siblings. The molecular genetic characterisation of these four patients with next-generation whole exome sequencing has been previously reported.^4^ ^5^ All the patients presented with progressive gait ataxia and they were severely disabled, requiring the use of a wheelchair for ambulation. Four patients (A, B, C and D) had significant peripapillary RNFL thickening outside the normal range for healthy controls (mean average thickness=100.3 μm, SD=1.8 μm). No patient had a significant refractive error that could be a confounding variable in the analysis of peripapillary RNFL thickness.

OD, right eye; OS, left eye; RNFL, retinal nerve fibre layer.

No patient had evidence of retinal hypermyelination. Retinal striations were observed around the optic discs in four patients (A, B, C and D) and OCT measurements showed significant generalised peripapillary RNFL thickening (see online supplementary figures S1–S4). Patient E had no fundus abnormalities, and the peripapillary RNFL thickness was within the normal range for healthy controls (see online supplementary figure S5).

Our case series has provided convincing evidence that abnormal retinal thickening is a common, although not universal, feature among patients with ARSACS-related phenotypes secondary to pathogenic *SACS* mutations. Significant peripapillary RNFL thickening has also been previously reported in eight patients harbouring confirmed pathogenic *SACS* mutations with different OCT imaging platforms to the one used in our study.^6–8^ Classical myelinated retinal nerve fibres were not observed, and the current body of evidence, at least among non-Québec patients, suggests that retinal ‘hypermyelination’ has been used inappropriately to describe the striated appearance of a thickened RNFL around the optic discs. The pathophysiological basis for this observation remains to be determined, but it is rather revealing that the sacsin protein localises to mitochondria. In a transgenic knock-out mouse model, depletion of the sacsin protein resulted in disruption of mitochondrial axonal transport and cerebellar neurones with aberrant dendritic morphologies.^9^ Although speculative, RNFL thickening in ARSACS could therefore be related to axoplasmic stasis within the long axons of the retinal ganglion cells as they converge to form the optic nerve in the anatomically constrained region of the lamina cribosa.

The investigation of a large multicentre cohort of patients with ARSACS will be needed to determine whether the degree of RNFL thickening correlates with disease severity and progression or whether there are any specific genotype–OCT correlations. The molecular genetic basis of inherited ataxia and spastic paraplegia syndromes is highly heterogeneous—a situation that poses a number of diagnostic challenges in neurology clinics. In Friedreich's ataxia, which is the most common form of autosomal recessive ataxia, variable reduction in RNFL thickness has been reported among visually asymptomatic patients.^10^ In a recent OCT study, we found a normal RNFL profile among patients harbouring *SPG4* mutations, which account for ∼40% of all autosomal-dominant cases of hereditary spastic paraplegia.^11^ A dilated fundus examination and OCT imaging should therefore be considered in patients with unexplained multisystem neurological disease. When present, retinal striations and RNFL thickening raise the distinct possibility of ARSACS, and *SACS* genetic screening should be considered.

## References

[1] Engert JC Berube P Mercier J ARSACS, a spastic ataxia common in northeastern Quebec, is caused by mutations in a new gene encoding an 11.5-kb ORF. Nat Genet 2000;24:120–5. 10655055 10.1038/72769

[2] Takiyama Y Sacsinopathies: sacsin-related ataxia. Cerebellum 2007;6:353–9. 17853117 10.1080/14734220701230466

[3] Garcia-Martin E Pablo LE Gazulla J Retinal nerve fibre layer thickness in ARSACS: myelination or hypertrophy? Br J Ophthalmol 2013;97:238–41. 23077228 10.1136/bjophthalmol-2012-302309 PMC3582091

[4] Pyle A Griffin H Yu-Wai-Man P Prominent sensorimotor neuropathy due to SACS mutations revealed by whole-exome sequencing. Arch Neurol 2012;69:1351–4. 22751902 10.1001/archneurol.2012.1472

[5] Pyle A Griffin H Duff J Late-onset sacsinopathy diagnosed by exome sequencing and comparative genomic hybridization. J Neurogenet 2013;27:176–82. 24180463 10.3109/01677063.2013.831094 PMC4038496

[6] Pablo LE Garcia-Martin E Gazulla J Retinal nerve fiber hypertrophy in ataxia of Charlevoix-Saguenay patients. Mol Vis 2011;17:1871–6. 21850161 PMC3144729

[7] Desserre J Devos D Sautiere BG Thickening of peripapillar retinal fibers for the diagnosis of autosomal recessive spastic ataxia of Charlevoix-Saguenay. Cerebellum 2011;10: 758–62. 21597885 10.1007/s12311-011-0286-x

[8] Nethisinghe S Clayton L Vermeer S Retinal imaging in autosomal recessive spastic ataxia of Charlevoix-Saguenay. Neuro-Ophthalmology 2011;35:197–201.

[9] Girard M Lariviere R Parfitt DA Mitochondrial dysfunction and Purkinje cell loss in autosomal recessive spastic ataxia of Charlevoix-Saguenay (ARSACS). Proc Natl Acad Sci USA 2012;109: 1661–6. 22307627 10.1073/pnas.1113166109 PMC3277168

[10] Fortuna F Barboni P Liguori R Visual system involvement in patients with Friedreichs ataxia. Brain 2009;132:116–23. 18931386 10.1093/brain/awn269

[11] Guthrie G Pfeffer G Bailie M The neurological and ophthalmological manifestations of SPG4-related hereditary spastic paraplegia. J Neurol 2012;260:906–9. 23238845 10.1007/s00415-012-6780-3 PMC3590400

